# *Lithothamnion muelleri* Controls Inflammatory Responses, Target Organ Injury and Lethality Associated with Graft-*versus*-Host Disease in Mice

**DOI:** 10.3390/md11072595

**Published:** 2013-07-18

**Authors:** Barbara M. Rezende, Priscila T. T. Bernardes, Carolina B. Resende, Rosa M. E. Arantes, Danielle G. Souza, Fernão C. Braga, Marina G. M. Castor, Mauro M. Teixeira, Vanessa Pinho

**Affiliations:** 1 Laboratory of Resolution of Inflammatory Response, Department of Morphology, Institute of Biological Sciences, Federal University of Minas Gerais, Belo Horizonte, 31270-901, Brazil; E-Mails: babirez@hotmail.com (B.M.R.); priscilattb@yahoo.com.br (P.T.T.B.); carolbresende@gmail.com (C.B.R.); marinacastor@gmail.com (M.G.M.C.); 2 Laboratory of Experimental Neuro-Immunopathology, Department of Pathology, Institute of Biological Sciences, Federal University of Minas Gerais, Belo Horizonte, 31270-901, Brazil; E-Mail: rosa@icb.ufmg.br; 3 Host-Microbes Interaction Laboratory, Department of Microbiology, Institute of Biological Sciences, Federal University of Minas Gerais, Belo Horizonte, 31270-901, Brazil; E-Mail: souzadg@gmail.com; 4 Department of Pharmaceutical Products, Faculty of Pharmacy, Federal University of Minas Gerais, Belo Horizonte, 31270-901, Brazil; E-Mail: fernao@netuno.lcc.ufmg.br; 5 Immunopharmacology, Department of Biochemistry and Immunology, Institute of Biological Sciences, Federal University of Minas Gerais, Belo Horizonte, 31270-901, Brazil; E-Mail: mmtex.ufmg@gmail.com

**Keywords:** algae, chemokine, cytokine, GVHD, inflammation

## Abstract

*Lithothamnion muelleri* (Hapalidiaceae) is a marine red alga, which is a member of a group of algae with anti-inflammatory, antitumor, and immunomodulatory properties. The present study evaluated the effects of treatment with *Lithothamnion muelleri* extract (LM) in a model of acute graft-*versus*-host disease (GVHD), using a model of adoptive splenocyte transfer from C57BL/6 donors into B6D2F1 recipient mice. Mice treated with LM showed reduced clinical signs of disease and mortality when compared with untreated mice. LM-treated mice had reduced tissue injury, less bacterial translocation, and decreased levels of proinflammatory cytokines and chemokines (interferon-γ (IFN-γ), tumor necrosis factor-α (TNF-α), chemokine (C-C motif) ligand 2 (CCL2), chemokine (C-C motif) ligand 3 (CCL3) and chemokine (C-C motif) ligand 5 (CCL5)). The polysaccharide-rich fraction derived from LM could inhibit leukocyte rolling and adhesion in intestinal venules, as assessed by intravital microscopy. LM treatment did not impair the beneficial effects of graft-*versus*-leukaemia (GVL). Altogether, our studies suggest that treatment with *Lithothamnion muelleri* has a potential therapeutic application in GVHD treatment.

## 1. Introduction

Allogeneic haematopoietic stem cell transplantation (HSCT) remains the major curative therapy for several haematological diseases [1,2]. The main complication from this therapy is graft-*versus*-host disease (GVHD), an immunological disorder with high morbidity and mortality [1,2]. The pathogenesis of GVHD is associated with immunocompetent donor cells that become activated upon recognition of antigens from the immunocompromised host and subsequently reject target host organs such as the lung, liver, and intestine [1,3,4].

Steroids such as methylprednisolone or prednisone are the first-line treatment for GVHD because they have broad anti-inﬂammatory and lymphocytotoxic effects [5,6]. However, steroid-refractory GVHD patients are at high risk of death from the disease or related complications, and there is no standard treatment strategy for these patients. Currently, some drugs that primarily interfere with the activation and signaling pathways of T cells, antigen-presenting cells, B cells, and NK cells, such as sirolimus and alemtuzumab, have been employed as second-line treatment for acute graft-*versus*-host disease [6,7,8]. Other drugs, such as inﬂiximab and etanercept, act against pro-inflammatory cytokines or their receptors to modify inflammatory responses [6,7,8]. However, most of the available treatments only induce clinical benefits in a limited subset of patients [5]. Few studies have investigated complementary medicine for the treatment of GVHD.

Recent evidence has indicated that exacerbated immune responses and associated complications can be alleviated with the use of natural products from marine species, including compounds isolated from marine algae [9]. Given the relevance of these findings, the evaluation of biological activities of algae extracts and derived compounds is a valid strategy for drug development.

*Lithothamnion muelleri* Lenormand ex Rosanoff (Hapalidiaceae) is a red seaweed found in Brazilian coastal areas and is rich in minerals like calcium carbonate and sulphated polysaccharides, and is potentially bioactive [10,11,12]. We have recently reported the reduction of leukocyte rolling induced by polysaccharide-rich fractions from *L. muelleri* [12]. Moreover, some studies have demonstrated the biological effects of other algae of the genus *Lithothamnion*, including the inhibition of polyp formation and inflammation in the gastrointestinal tract [13], and protection against formation of liver tumors in mice fed with a high-fat diet [14]. The present study suggests that treatment with *Lithothamnion muelleri* extract (LM) may be a potential new therapy to control inflammatory responses, tissue injuries, and lethality associated with graft-*versus*-host disease. 

## 2. Results and Discussion

### 2.1. LM Treatment Reduced Intestinal and Hepatic Injury in Mice Subjected to GVHD

To verify the possible therapeutic properties of *L. muelleri* in GVHD, this disease was induced by transfer of splenocytes from C57BL/6J donors to B6D2F1 mice. Mice subjected to GVHD and not treated were considered the GVHD group. The LM group was subjected to GVHD and treated with 1% LM in the diet (w/w). In a dose-response experiment, the dose of 1% LM in the diet (w/w) was found to be optimal for inhibiting GVHD and was chosen for subsequent experiments (Supplementary Figure S1). 

Intestine and liver sections were evaluated at days 3, 10, and 20 after transplantation. In the GVHD group, at days 3 and 10 there was mild histopathological injury associated with oedema, congestion of the lamina propria, and increased cellularity associated with focal areas of villous enlargement. Additionally, there was mild focal infiltration in the muscular and serous layers. At these time-points, there were no differences in mice subjected to GVHD, regardless of LM treatment (data not shown). 

**Figure 1:**
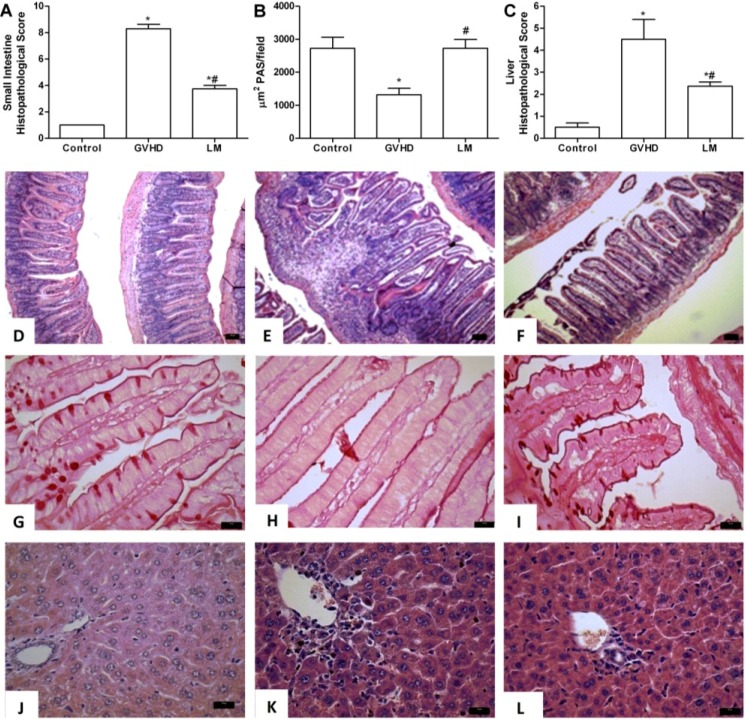
*Lithothamnion muelleri* extract (LM) treatment reduces intestinal and hepatic injury in mice subjected to GVHD. GVHD was induced by the adoptive transfer of splenocytes from C57BL/6J donors to B6D2F1 mice. Mice that received syngeneic (B6D2F1) splenocytes did not develop disease and were considered the Control group. LM (1% in the diet, w/w) was offered in the diet of recipient mice from one day before transplantation until the experimental endpoint. At day 20 after transplantation, the mice were sacrificed and the jejunum-ileum and liver tissues were sampled for histopathological analysis (**A** and **C**). The numbers of PAS-positive goblet cells counted at 40×/field were determined from four fields per intestine section (**B**). **D**–**F**, histological aspects of H&E-stained small intestine sections in Control, GVHD, and LM-treated mice, respectively. Scale bar, 50 μm for all panels. **G**–**I**, intestine sections with PAS-positive goblet cells from Control, GVHD, and LM treated mice, respectively. **J**–**L**, histological aspects of H&E-stained liver sections from Control, GVHD, and LM treated mice, respectively. Scale bar, 20 μm for all panels. The results are presented as the mean ± SEM (*n* = 6); * and ^#^, *p* < 0.05 when compared with the Control and GVHD groups, respectively.

At day 20 after transplantation, there was partial loss of organ architecture, increased cellularity, oedema, and congestion in the intestine of animals that had been subjected to GVHD. Severe degenerative changes, ulcerations of the mucosa, and areas of focal necrosis in the muscular and serous layers were also observed at this time-point (Figure 1A,E). LM treatment signiﬁcantly ameliorated the overall pathological scores. Changes in organ architecture were decreased and were characterized by superﬁcial and rare erosions in the mucosa, reduced inﬂammatory inﬁltration and oedema in the lamina propria, and preserved muscular and serous layers (Figure 1A,F).

The mucus layer is considered to be the first line of defense between bacteria in the lumen and host cells, and also serves as the initiation surface for host-microbe interactions [15,16,17]. The major components of mucus are mucins, a family of heavily glycosylated proteins [15,16,17]. As observed in Figure 1B,H, there was reduction in the number of goblet cells in the GVHD group, as determined by Periodic Acid-Schiff (PAS) staining, which acts as a marker for mucin. In contrast, LM treatment maintained the number of goblet cells at a level similar to that of the control group (Figure 1B,G,I), which might have contributed to the intestinal protection afforded by LM. 

There were few alterations in the liver at day three after transplantation in the GVHD group (data not shown). At day 10, the GVHD group presented with discreet oedema and increased cellularity in the parenchyma and periportal areas. The latter parameters were not modified by LM treatment (data not shown). However, at day 20 after transplantation, there was signiﬁcant injury throughout the liver parenchyma (Figure 1C,K). Increased inflammatory infiltration was observed mainly in the periportal areas and vasodilatation, hepatocyte necrosis, and diffuse vacuolisation were also observed (Figure 1K). There was signiﬁcant preservation of hepatic tissue with mild oedema and decreased inﬂammatory cell accumulation in the LM group (Figure 1C,L). Other *Lithothamnion* species, including *L. calcareum* [13] and a mixture of *L. calcareum* and *L. coraloides* [18] have also been shown to reduce intestinal and hepatic injury in other models of inflammation. Of interest, 50% of patients who developed GVHD also develop hepatic and intestinal injuries [19]. Therefore, LM-induced protection against severe liver and intestinal damage may be eventually exploited in the clinical setting.

### 2.2. LM Treatment Reduced Bacterial Translocation and Macrophage Accumulation in Mice Subjected to GVHD

**Figure 2:**
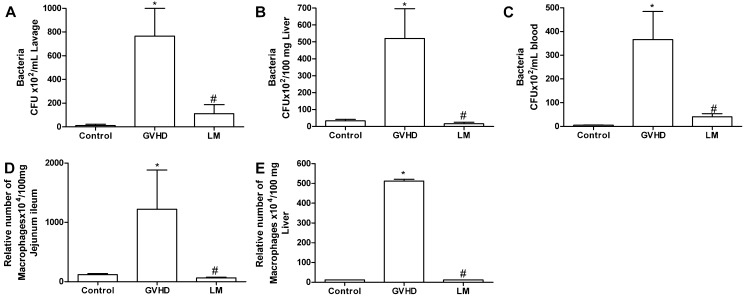
*Lithothamnion muelleri* extract (LM) treatment inhibits bacterial translocation and macrophage accumulation in target organs and blood of mice subjected to GVHD. GVHD was induced by the transfer of splenocytes from C57BL/6J donors to B6D2F1 mice. Mice that received syngeneic (B6D2F1) splenocytes did not develop disease and were considered the Control group. LM (1% in the diet, w/w) was offered in the diet of recipient mice from one day before transplantation until the experimental endpoint. At day 20 after transplantation, the mice were sacrificed and the bacterial translocation to the peritoneal cavity (**A**), blood (**B**), and liver (**C**) were quantified. Macrophages were quantified in the jejunum-ileum (**D**) and liver (**E**) by enzymatic methods. The results are presented as the mean ± SEM (*n* = 6); * and ^#^, *p* < 0.05 when compared with the Control and GVHD groups, respectively.

Differences between the treated and untreated groups were more evident at day 20 after transplantation. Therefore, all subsequent evaluations were carried out at this time-point. Although there is much liver damage [20], clinical and experimental evidence suggests that the intestine is the main organ related to the pathophysiology of GVHD and contributes significantly to causing systemic disease [21]. Intestinal injury is associated with bacterial translocation to the liver and subsequently into the bloodstream, leading to sepsis [22,23,24,25]. At day 20 after transplantation, high number of bacteria was observed in the peritoneal lavage fluid from the GVHD group (Figure 2A). Bacterial levels were also increased in the liver and blood of animals subjected to GVHD (Figure 2B,C). In contrast, fewer bacteria were observed in the peritoneal cavity, liver, and blood of LM-treated mice that were subjected to GVHD, which was reflected by the overall amelioration of the disease (Figure 2A–C). This is an important ﬁnding as endotoxin has been identiﬁed as a major mediator of intestinal and systemic inﬂammation in murine GVHD [23,26]. Moreover, as observed previously, there was a reduction of the intestinal mucus layer in the GVHD group and this layer was significantly preserved after LM treatment (Figure 1B,H,I). Mucin degradation by bacteria often occurs during the initial stages of some intestinal diseases and can modify the protection of the host mucosal surfaces [15,16,17,27]. In this context, the preservation of the mucus layer in LM-treated mice might have contributed to effective intestinal protection and might also have been associated with reduced bacterial translocation. In fact, bacterial translocation and sepsis are important causes of death in GVHD patients [25]. 

There were high number of macrophages in the intestine and liver of mice subjected to GVHD (Figure 2D,E). In LM-treated mice, macrophage infiltration in the jejunum ileum and liver was reduced (Figure 2D,E). This result is relevant because macrophages are important for GVHD development. These cells are recruited to target organs, have phagocytic functions, participate in antigen presentation to T lymphocytes, and produce inflammatory mediators, including reactive oxygen species, chemokines, and cytokines [28]. Therefore, the reduction of macrophages observed in the LM group may be associated with protection from GVHD.

### 2.3. LM Treatment Reduced the Production of Chemokines and Proinflammatory Cytokines in the Intestines and Livers of GVHD Mice

**Figure 3:**
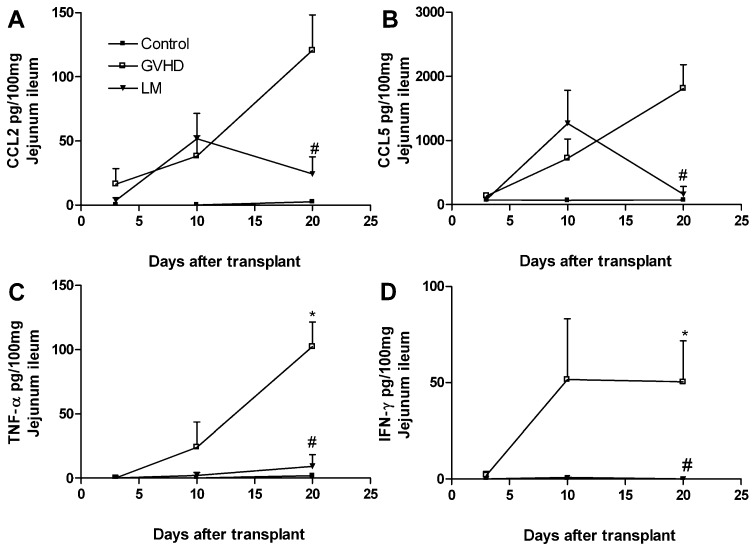
*Lithothamnion muelleri* extract (LM) treatment reduces the concentrations of cytokines and chemokines in the small intestine of mice subjected to GVHD. GVHD was induced by the transfer of splenocytes from C57BL/6J donors to B6D2F1 mice. Mice that received syngeneic (B6D2F1) splenocytes did not develop disease and were considered the Control group. LM (1% in the diet, w/w) was offered in the diets of recipient mice from 1 day before transplantation until the experimental endpoint. At 20 days after transplantation, the mice were sacrificed and the concentrations of CCL2 (**A**), CCL5 (**B**), TNF-α (**C**) and IFN-γ (**D**) in the intestinal homogenates were evaluated by Enzyme Linked Immuno Sorbent Assay (ELISA). The results are shown as the mean ± SEM (*n* = 5); * and ^#^, *p* < 0.05 when compared with the Control and GVHD groups, respectively.

GVHD is associated with increased levels of pro-inflammatory mediators in the target organs. Herein, there were increased levels of CCL2, CCL5, TNF-α, and IFN-γ in the intestine of mice subjected to GVHD, mainly at 20 days after transplantation (Figure 3). LM treatment substantially decreased the production of cytokines and chemokines at that time-point. 

**Figure 4:**
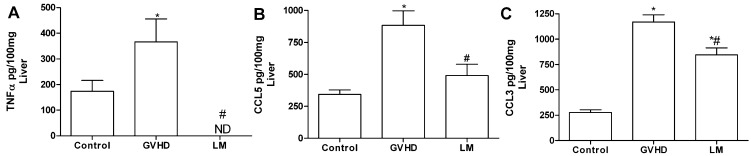
*Lithothamnion muelleri* extract (LM) treatment reduces the concentrations of cytokines and chemokines in the liver of mice subjected to GVHD. GVHD was induced by the transfer of splenocytes from C57BL/6J donors to B6D2F1 mice. Mice that received syngeneic (B6D2F1) splenocytes did not develop disease and were considered the Control group. LM (1% in the diet, w/w) was offered in the diets of recipient mice from one day before transplantation until the experimental endpoint. At 20 days after transplantation, the mice were sacrificed and the concentrations of CCL3 (**A**), CCL5 (**B**), and TNF-α (**C**) in the liver homogenates were evaluated by ELISA. The results are shown as the mean ± SEM (*n* = 5); * and ^#^, *p* < 0.05 when compared with the Control and GVHD groups, respectively.

There were also increased levels of the chemokines CCL3 and CCL5 and of the cytokine TNF-α in the liver of mice subjected to GVHD at day 20 after transplantation (Figure 4), although no significant differences was observed between the levels of CCL2 and IFN-γ in the liver of LM-treated and untreated mice subjected to GVHD (Supplementary Figure S2). LM treatment completely inhibited TNF-α production and reduced CCL5 and CCL3 production (Figure 4A–C). The increased macrophage accumulation in the intestine and liver of GVHD mice observed previously in this work might be secondary to the production of CCL2, CCL3, and CCL5, which were observed in GVHD group, as these chemokines are known chemoattractants of macrophages and T cells. Macrophages can also produce such cytokines and chemokines to perpetuate the inflammatory process observed in GVHD [29,30,31,32,33,34,35,36,37]. Previously, our group reported that intestinal CCL2 levels were increased from the early stages of GVHD [35]. Moreover, the interaction of CCL2 with its receptor facilitates the activation and migration of CD8^+^ T lymphocytes to GVHD target organs [38]. CCL3 is another important chemokine in GVHD. Recent studies have demonstrated that the absence of CCL3 in the donor cells or the pharmacological blockade of the CCL3 receptor reduces GVHD-associated mortality and target organ damage, reduces the accumulation of CD4^+^ and CD8^+^ T lymphocytes in the spleen and intestine, and inhibits the recruitment of macrophages to the intestines [29,34]. Furthermore, the neutralisation of CCL3 was associated with reduced levels of CCL5 in the intestines of GVHD mice, which suggested that CCL3 could modulate CCL5 levels in this organ [34]. The absence of the CCL5 receptor was also associated with improved survival, reduced clinical disease, reduced levels of TNF-α and IFN-γ, and the reduced recruitment of inflammatory cells such as T lymphocytes and mononuclear cells to the GVHD target organs [32]. IFN-γ and TNF-α are well-known to have relevant roles in GVHD [19]. Increased levels of these cytokines precede clinical GVHD symptoms and are associated with a systemic syndrome of weight loss, diarrhea, skin changes, and high mortality [39,40,41,42]. We also observed increased levels of these cytokines at day 10 after transplantation in GVHD mice; this finding coincided with the onset of clinical disease and subsequent mortality as seen below. On the other hand, LM-treated GVHD mice presented with reduced levels of IFN-γ and TNF-α, which might have contributed to the protection of the target organs and reduced GVHD-associated mortality. In this context, we suggest that the reduced levels of chemokines and cytokines that are associated with reduced macrophage accumulation might have contributed to the reduced inflammatory responses in LM-treated GVHD mice.

### 2.4. Polysaccharide-Rich Fraction of *L. muelleri* Inhibited Leukocyte-Endothelial Cell Interactions in the Intestinal Microvasculature of GVHD Mice

**Figure 5:**
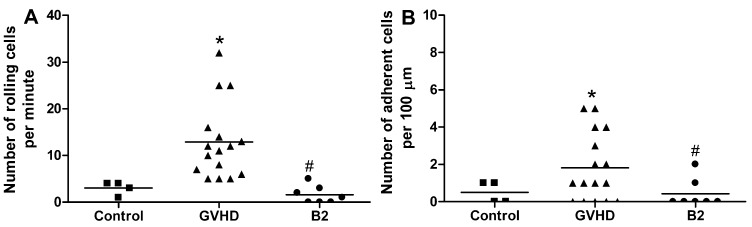
Treatment with a polysaccharide fraction of *Lithothamnion muelleri* extract (LM) decreases the number of rolling and adherent leukocytes in the mesenteric vasculature of mice subjected to GVHD. GVHD was induced by the transfer of splenocytes from C57BL/6J donors to B6D2F1 mice. Mice that received syngeneic (B6D2F1) splenocytes did not develop disease and were considered the Control group. At day 10 after transplantation, the mice were anesthetized and intestinal venules (±40 μm) were selected in which to count the numbers of rolling and adherent leukocytes by intravital microscopy. Mice that received C57BL/6J splenocytes were treated either with the vehicle or with a polysaccharide fraction of LM (B2, 100 mg/kg) at 30 min before intravital microscopy. (**A**) Number of rolling cells/minute; (**B**) Number of adherent cells/100 μm is presented as the mean ± SEM (*n* = 7–16). Control group (■), GVHD group (▲) and B2 group (●). * and ^#^, *p* < 0.05 when compared with the Control and GVHD groups, respectively.

*L.* *muelleri* is composed mainly of calcium carbonate (CaCO_3_), which represents 80%–90% of its biomass [43]. Recent studies with *Lithothamnion* species have demonstrated the capacity of these algae to suppress intestinal polyp formation and reduce gastrointestinal inflammation induced in animals fed a high-fat diet. In these studies, the mechanism behind the protective effect of algae is associated with the activity of their mineral constituents, including CaCO_3_ [13,44]. Thus, the effects of CaCO_3_ on GVHD were investigated and compared to LM treatment (Supplementary Figure S3). To assess this, GVHD was induced and mice were given normal chow (GVHD group) or treated with 1% LM (LM group) or with 0.9% CaCO_3_ (CaCO_3_ group) in the diet. CaCO_3_ did not recapitulate the protective mechanisms of LM treatment in GVHD. Indeed, weight loss and clinical score were similar mice given CaCO_3_ and the control group, whereas LM treatment reduced disease significantly in the same experiment. Therefore, other constituents of LM appear to be associated with GVHD protection. Some polysaccharide-rich fractions have been recently obtained from the species and characterized by Soares *et al.* (2012) [12]. Here, intravenous injections of LM fractions reduced lipopolysaccharide-induced leukocyte rolling in mice by approximately 90% [12]. Currently, it is known that sulphated polysaccharides from seaweed have important biological properties such as anticoagulant, antioxidant, antitumor, antiviral, and anti-inflammatory activities [45,46]. Thus, we assessed whether LM polysaccharides (B2 fraction) could interfere directly with the initial stages of leukocyte migration to the GVHD target organs (B2 group). We performed intravital microscopy on the intestinal postcapillary venules of animals subjected to GVHD at day 10 after transplantation. The mice were treated with B2 at 30 min before the microscopic analysis. As seen in Figure 5, B2 treatment reduced the numbers of both rolling and mesenteric venule-adherent leukocytes. LM fractions-induced decreases in leukocyte rolling and adhesion could explain the reduced levels of inflammatory infiltration into the evaluated target organs as well as the control of GVHD-associated inflammatory responses. 

### 2.5. LM Treatment Reduced Clinical Disease and Mortality in Mice Subjected to GVHD

**Figure 6:**
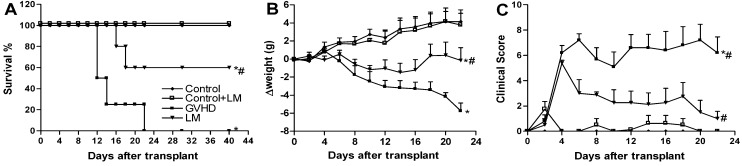
*Lithothamnion muelleri* extract (LM) treatment decreases death, weight loss, and clinical signs in mice subjected to GVHD. GVHD was induced by the transfer of splenocytes from C57BL/6J donors to B6D2F1 mice. Mice that received syngeneic (B6D2F1) splenocytes did not develop disease and were considered the Control group. LM (1% in the diet, w/w) was offered in the diets of recipient mice from one day before transplantation until the experimental endpoint. After the induction of GVHD, the mice were evaluated every two days for survival (**A**), body weight (**B**), and clinical scoring (**C**). The results are shown as the mean ± SEM and the numbers of animals were as follows: Control group (♦), *n* = 6; Control + LM group (□), *n* = 6; GVHD group (■), *n* = 7 and LM group (▼), *n* = 7. * and ^#^, *p* < 0.05 when compared with the Control and GVHD groups, respectively.

Some hallmark features of GVHD include mortality, weight loss and clinical signs such as a loss of skin integrity and fur texture and diarrhea and occult blood in the faeces [47,48]. We therefore evaluated whether LM treatment could prevent GVHD-associated mortality. As shown in Figure 6A, all B6D2F1 mice that received splenocytes from C57BL/6J mice and were not treated (GVHD group) died before day 22. This mortality was associated with significant weight losses and high clinical scores. The control group did not develop GVHD and thus all mice were alive at the end of the experiment. The remaining mice were killed on day 40, which was the last day of observation. B6D2F1 mice that received splenocytes from C57BL/6J mice and were treated with LM (LM group) had prolonged survival and reduced, both, weight losses and clinical scores (Figure 6A–C). Of note, LM-treated animals that were not subjected to GVHD induction (Control + LM group) had the same survival rate, weight gain, and clinical score as the control animals (Figure 6); these data indicate that treatment with *L. muelleri* alone did not affect the analyzed parameters. These data are consistent with our previous results that demonstrated reduced target organ injury and lower GVHD-associated inflammatory responses, which ultimately led to significantly improved survival rates in LM-treated mice.

Different animal models of GVHD have been used since the 1970s. These models are useful for understanding the mechanisms involved in GVHD-associated inflammatory responses and for the development of new therapeutic strategies [49]. The induction of acute GVHD in mice involves the transplantation of splenocytes and/or bone marrow cells and can vary, depending on the dose of radiation used to ablate the immune cells of the receptor. In partial body irradiation, also known as the non-myeloablative regimen, it is not necessary to completely reconstitute the bone marrow. After transplantation, mice demonstrate chimerism, in which most of the cells are donor-derived [37,42]. The total body irradiation model, also known as the myeloablative regimen, requires an infusion of bone marrow precursor cells for replenishment [37,42]. When combined with the transplantation of bone marrow cells and splenocytes, models that employ total body irradiation result in more severe disease. The irradiation dose is proportional to the degrees of tissue damage and subsequent cytokine production that are observed during the conditioning regimen, and these factors can influence the development of GVHD [42]. Thus, to confirm the beneficial effects of LM treatment in graft-*versus*-host disease and to establish relevance to the human disease, we tested LM treatment in mice that had been subjected to total body irradiation as described in the Materials and Methods. LM treatment was also protective in this model (Supplementary Figure S4), which highlights the effectiveness and relevance of LM treatment that we have observed.

### 2.6. Treatment with LM Decreased GVHD but Did Not Interfere with GVL Response

**Figure 7:**
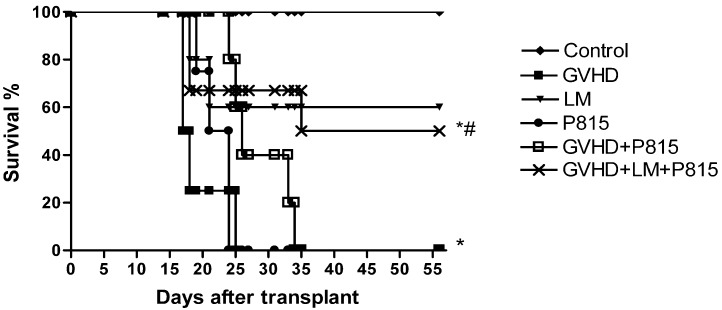
*Lithothamnion muelleri* extract (LM) treatment does not interfere with GVL in mice subjected to GVHD. GVHD was induced by the transfer of splenocytes from (C57BL/6J) donors to B6D2F1 mice. GFP^+^ P815 cells were injected i.v. into B6D2F1 recipients on day 0 of transplantation. Mice that received only syngeneic (B6D2F1) splenocytes did not develop disease and were considered the Control group. The P815 group received only GFP^+^ P815 cells, and the other groups received splenocytes from (C57BL/6J) donors as well as GFP^+^ P815 cells. LM(1% in the diet w/w) was offered in the diets of recipient mice from one day before transplantation until the experimental endpoint. After the induction of GVHD, the mice were evaluated every two days for survival. The results are shown as the mean ± SEM. Control group (♦), *n*=6; GVHD group (■), *n*=6; LM group (▼), *n*=6; P815 group (●), *n*=6; GVHD + P815 group (□), *n*=7 and GVHD + LM + P815 group (**×**), *n*=7. * and ^#^, *p* < 0.05 when compared with mice that received no tumor cells and mice that received tumor cells but were not subjected to GVHD, respectively.

Several therapies that are effective in preventing GVHD usually lead to reduced graft-*versus*-leukaemia activity (GVL), which is the ability of donor-derived infused lymphocytes in a haematopoietic stem cell-transplanted patient to react against leukemic cells, invalidating their effective applications [50,51,52,53]. Graft-*versus*-host disease typically correlates with desired GVL reactivity [54,55,56]. Thus, we assessed whether LM treatment could interfere with beneficial graft-*versus*-leukaemia response. To determine this, GVHD was induced by a transfer of allogeneic splenocytes from C57BL/6J to B6D2F1 mice. The control group received a transfer of syngeneic splenocytes from B6D2F1 mice. Mice that were subjected to GVHD were treated or not with LM (LM group and GVHD group, respectively). To verify the GVL response, the GVHD mice were injected with 10^4^ GFP^+^ P815 cells immediately after splenocytes transplantation at day 0 and were not treated (GVHD + P815 group). One group of GVHD mice that received GFP^+^ P815 cells had been treated with LM, which was offered in the diet from one day before transplant until the end of the experiment (GVHD + LM + P815 group). Finally, to verify the viability of the P815 cells, one group of mice received only the P815 injection and was not subjected to GVHD (P815 group). Mice were monitored every two days for survival. Mice in the control group did not develop GVHD and there were no deaths in this group. The mice that had been subjected to GVHD and were not treated with LM (GVHD group) died until 25 days after transplantation. The mice that received only tumor cells (P815 group) presented with 100% lethality at day 24 after transplantation, which indicated the viability of the tumor cells. GVHD mice that received P815 cells remained alive until 35 days after transplantation. Thus, the GVHD mice that received P815 cells died from GVHD rather than from the tumor. LM treatment reduced the severity of GVHD without interfering with the beneficial responses of the allogeneic cells against the tumor because mice that received both splenocytes and tumor cells (GVHD + LM + P815 group) presented with a similar survival rate as the LM-treated mice that received only splenocytes (LM group) (Figure 7).

## 3. Experimental Section

### 3.1. Mice

Eight- to 12-week-old C57BL/6 and B6D2F1 (C57BL/6 × DBA/2) mice were obtained from the Centro de Bioterismo (UFMG, Belo Horizonte, Brasil) and housed under standard conditions in a temperature-controlled room (23 ± 1 °C) on an automatic, 12-h light/dark cycle. Mice had free access to commercial chow and water. All animal care and experimental procedures were performed in accordance with the local Institutional Committee for Animal Care and Use (it is approved by protocol number: 120/09). 

### 3.2. Algal Material

Extracts of *L. muelleri* were donated by the company Phosther Algamar (Belo Horizonte, Minas Gerais, Brazil) as a whitish granulate known as marine mineral concentrate. The granulate was produced by washing the seaweed sequentially with tap water and distilled water to remove salt and all visible epiphytes, following by crushing in a ball mill and drying in a ventilated oven. The species was identified by Maria Carolina M. de O. Henriques, Instituto Biodiversidade Marinha, Rio de Janeiro, Brazil.

### 3.3. Polysaccharide-Rich Fraction

The polysaccharide-rich fraction (B2) was obtained as previously described [12]. Briefly, portions of the granulate were submitted to extraction with water at 60 °C for 6 h under mechanical stirring, using the proportion of 5% (w/v) granulate in distilled water. After filtration, the remaining residue was reextracted sequentially with 1% and 2% (w/v) Na_2_CO_3_ solution for 2 h at 60 °C (5% w/v material/solvent extractor), originating fractions B1 and B2 (final fraction used in this work), respectively. In the sequence, the extracts were concentrated in a rotatory evaporator and ethanol was added for polysaccharide precipitation. The precipitates were then dialyzed against water through a cellulose membrane and water was removed using a centrifugal vacuum concentrator. The chemical composition of B1 and B2 fractions has been previously investigated [12]. As stated in the mentioned publication, the chemical analysis of these fractions suggested related compositions, with similar contents of total carbohydrates [(40.0 ± 1.0)% and (38.8 ± 0.9)%, respectively, for B1 and B2] and uronic acid [(4.5 ± 0.1)% and (4.6 ± 0.1)%], but differences in the concentrations of sulfate [(3.6 ± 0.9)% and (18.0 ± 1.0)%] and total proteins [(6.0 ± 1.0)% and (2.3 ± 0.1)%].

### 3.4. Induction of GVHD

#### 3.4.1. Partial Body Irradiation

GVHD was induced by i.v. injection of 3 × 10^7^ splenocytes from syngeneic (B6D2F1) or semiallogeneic (C57BL/6) donors into recipient B6D2F1 mice that had been irradiated with a single 4 Gy dose of partial body irradiation (source CO^60^) 2 days prior to transplantation, as described previously [34,35,36]. 

#### 3.4.2. Total Body Irradiation

Recipient B6D2F1 mice were irradiated with 8 Gy total body irradiation (source CO^60^) in 2 doses at a 2 h interval to minimize gastrointestinal toxicity, followed by an i.v. infusion of 3 × 10^7^ splenocytes and 1 × 10^7^ bone morrow cells from syngeneic (B6D2F1) or semiallogeneic (C57BL/6) donors. In this model, due to the toxicity of total body irradiation, the recipient mice received an oral suspension of ciprofloxacin (70 mg/L) in their drinking water from 1 day before to 15 days after the transplantations.

#### 3.4.3. Cell Preparation

Bone marrow suspensions were prepared by flushing femurs with Roswell Park Memorial Institute medium (RPMI)-1640 medium that was supplemented with 10% fetal calf serum (FCS). Splenocytes were obtained by gently crushing spleens in complete medium to release the cells, which were then filtered to remove debris and washed twice in phosphate buffered saline (PBS) before injection. 

#### 3.4.4. Experimental Groups

B6D2F1 mice, which received splenocytes from B6D2F1 mice (B6D2F1 to B6D2F1), did not develop disease and were considered the control group. The GVHD, LM and CaCO_3_ groups received splenocytes from C57BL/6 mice (C57BL/6 to B6D2F1) and developed classic disease. 

### 3.5. Therapies

LM was administered at concentrations of 0.1%, 0.3%, and 1% w/w in the diet of the LM group from 1 day before transplantation until the experimental endpoint. For the dietary preparations, 100 g of crushed diet were combined with LM at 0.1%, 0.3%, or 1% w/w in 60 mL of filtered water to determine the dose response. The GVHD group received only crushed diet in 60 mL of filtered water. The CaCO_3_ group received a diet supplemented with 0.9% w/w of calcium carbonate, prepared similarly to the LM group. The effect of the LM polysaccharide-rich fraction was analyzed by intravital microscopy in mice that had been treated with a single dose of the fraction, 100 mg/kg in 200 μL of PBS containing diluted ethanol in a concentration of 5% (v/v), or 200 μL of vehicle, PBS containing diluted ethanol in a concentration of 5% (v/v), 30 min before the experiment.

### 3.6. Mortality Rate and Assessment of GVHD Clinical Score

Mice were monitored daily for survival after the transplants and were evaluated clinically with a standard scoring system that generated a composite GVHD score from the individual scores for weight loss, posture (hunching), activity, fur texture, skin integrity, diarrhea, and faecal occult blood. A clinical index was subsequently generated by the summation of the seven criteria scores (maximum index = 14), as described previously [34,35,36].

### 3.7. Histopathology

A set of experiments was conducted to quantify the histopathological parameters in the intestine and liver, which are important GVHD target organs. Tissue sections were processed for histological analysis as described previously [34] and were evaluated by a pathologist. A numerical value was attributed to the changes observed in the intestinal layers (mucosa, lamina propria, muscular, and serosa) or in the liver (degenerative alterations of parenchyma) were evaluated and each animal received a score that was generated by a summation of all observed changes (maximum index: 9 for intestine and 6 for liver) as described in Castor *et al.* [34]. Histopathological scores were determined for the samples that were obtained from mice at day 20 after the transplant, which corresponded to the clinical disease mortality phase in mice. The intestines and livers were removed for histopathological analysis from some mice at 24 h after irradiation and before transplantation to control for the consequences of irradiation to these organs. Signiﬁcant pathological changes were not detected at this time-point (data not shown).

### 3.8. Periodic Acid-Schiff (PAS) Staining

After the induction of GVHD, the mice were sacrificed at day 20 after transplantation. The intestines (jejunum-ileum) were processed for histological analysis as described previously [34] and were stained with Periodic Acid-Schiff. The numbers of PAS-positive goblet cells were counted in four fields per intestine at 40×/field, and a mean number/field was calculated for each sample. 

### 3.9. Quantiﬁcation of Macrophage Inﬁltration

The relative numbers of infiltrating macrophages in the intestine and liver were quantiﬁed by measuring the *N*-acetyl glucosaminidase (NAG) activity at day 20 after transplantation. A 100 mg portion of the small intestine was resuspended in 0.9% saline (4 °C) with 0.15 v/v Triton X-100 (Merck, Rahway, NJ, USA), homogenized, and centrifuged at 4 °C for 10 min at 1500 rpm. The supernatants were collected and assayed immediately for NAG activity at a 1:10 dilution as described previously [57].

### 3.10. Bacterial Translocation

At day 20 after transplantation, 100 μL of blood, 100 μL of peritoneal lavage fluid, and 100 mg of liver homogenate were plated onto Muller Hilton plates. These plates were incubated for 24 h at 37 °C and the numbers of bacterial colonies were counted and expressed as colony-forming unit (CFU).

### 3.11. Quantiﬁcation of Cytokines and Chemokines

The concentrations of cytokines and chemokines were quantiﬁed from intestinal or liver homogenates from animals at days 3, 10, and 20 after transplantation. The tissues were mixed with PBS that contained antiproteases (0.1 mM phenylmethanesulfonylfluoride (PMSF); 0.1 nM benzethonium chloride; 10 mM ethylenediamine tetraacetic acid (EDTA); 20 Kallikrein Inhibitor Units (KIU), aprotinin A) and 0.05% Tween 20. Next, the samples were centrifuged for 10 min at 10,000 rpm and 4 °C. Dilutions of the supernatants in PBS (1:4) were immediately analyzed by ELISA. The cytokines and chemokines concentrations were measured according to the manufacturer’s procedures (R & D Systems, Minneapolis, MN, USA) and the colorimetric reactions were analyzed with a spectrophotometer at a wavelength of 492 nm.

### 3.12. Intravital Microscopy

GVHD was induced in B6D2F1 mice by the transplantation of C57BL/6 splenocytes. At day 10 after transplantation, the mice were anesthetized and the intestinal venules were exposed in a perfusion system with warm bicarbonate-buffered saline (pH 7.4). For intravital microscopy analysis, mice that had received C57BL/6 splenocytes were treated with a single 200 μL dose of the vehicle (PBS-5% ethanol) or LM polysaccharide-rich fraction (B2, 100 mg/kg in 200 μL of PBS-5% ethanol) at 30 min before the intravital microscopic analysis. The fraction was administered just prior to the intravital procedure to prevent any changes to the local production of proinﬂammatory mediators. In the control group, B6D2F1 mice received B6D2F1 splenocytes. An intravital microscope (ECLIPSE 50i, Nikon, Japan) with a 20 objective lens was used to examine the mesenteric microcirculation. A digital camera (DS-Qi1MC, Nikon, Japan) was used to project the images onto a computer monitor, and the images were recorded for playback analysis with Nikon Imaging Software (Nikon, Kawasaki, Japan). The intestinal venules 40–60 μm were selected and the numbers of rolling and adherent leukocytes were determined off-line during the video-playback analysis. Rolling leukocytes were deﬁned as those cells that moved at a velocity less than that of the erythrocytes within a given vessel. The ﬂux of rolling cells was measured as the number of rolling cells that passed by a given point in the venule per minute. A leukocyte was considered to be adherent if it remained stationary for at least 30 s, and total leukocyte adhesion was quantiﬁed as the number of adherent cells in the intravascular space within an area of 100 μm.

### 3.13. GVL Induction

A P815 mouse mastocytoma cell line (H-2^d^; American Type Culture Collection, Manassas, VA, USA) that had been transduced with a lentiviral vector (elongation factor 1-GFP) was kindly provided by Anna C. Leal and Martin Bonamino (Instituto Nacional do Câncer, Rio de Janeiro, Brazil). This cell line was maintained in RPMI/10% FCS at 37 °C and 5% CO_2_ and was used for GVL (graft-versus-leukaemia) experiments *in vivo*. The above-described protocols for irradiation and GVHD induction were used. B6D2F1 recipients were injected i.v. with 10^4^ GFP^+^ P815 cells on day 0 of the transplantation experiment. After the induction of GVHD and transplantation of tumor cells, the mice were monitored every two days for survival. LM was offered in the diets of recipient mice from one day before transplantation until the experimental endpoint.

### 3.14. Statistical Analysis

Data in the text are expressed as the mean ± SEM. Comparisons between the groups were performed by ANOVA, followed by the Student Newman-Keuls *post hoc* analysis. A log-rank test was used to compare the relevant survival curves. Statistical signiﬁcance was set as *p* < 0.05, and all graphs and analysis were performed with GraphPad Prism 4 software (GraphPad Software Inc., San Diego, CA, USA).

## 4. Conclusions

The present study evaluated the effects of treatment with the alga *Lithothamnion muelleri* in the pathogenesis of acute graft-*versus*-host disease. In summary, we have demonstrated that LM treatment reduced intestinal and hepatic injuries, bacterial translocation, and macrophage accumulation after the transplantation of C57Bl/6 mice splenocytes into B6D2F1 mice. Additionally, LM treatment was accompanied with reduced production of pro-inflammatory mediators, including TNF-α, IFN-γ, CCL2, CCL3, and CCL5. Decreased leukocyte accumulation was associated with the anti-inflammatory effects of sulphated polysaccharides that were extracted from LM and which interfered with leukocyte-endothelium interactions in the intestinal venules as determined by intravital microscopy. Importantly, LM treatment resulted in reduced manifestations of GVHD in both animal models (partial or total body irradiation) as determined by reductions in the lethality rates and clinical scores. Finally, LM treatment did not interfere with the beneﬁcial effects of the graft against the GFP^+^ P815 leukaemia cell line. Thus, the results presented herein reveal *Lithothamnion muelleri* as a new candidate with potential therapeutic applications for the treatment of graft-*versus*-host disease.

## Supplementary Files

Supplementary File 1 Supplementary Information (PDF, 135 KB)
