# Supplementary material for: Lithothamnion muelleri Controls Inflammatory Responses, Target Organ Injury and Lethality Associated with Graft-versus-Host Disease in Mice
Source: Mar Drugs. 2013 Jul 18;11(7):2595–615. doi: 10.3390/md11072595 (PMC3736440; doi:10.3390/md11072595)

# Supplementary Information

**Figure S1.** Dose response of *Lithothamnion muelleri* extract (LM) in mice subjected to GVHD. GVHD was induced by the transfer of splenocytes from C57BL/6J donors to B6D2F1 mice. Mice that received syngeneic splenocytes did not develop disease and were considered the Control group. LM (in the diet, w/w) was offered at concentrations of 0.1%, 0.3% and 1% in the diets of recipient mice from 1 day before transplantation until the experimental endpoint. After the induction of GVHD, the mice were evaluated every 2 day for survival (**A**) and GVHD clinical scores (**B**). The remaining mice were sacrificed on day 40, which was the last day of observation. The results are shown as the mean ± SEM. Control group (♦), $n$ = 6; GVHD group (■), $n$ = 7, LM 0.1% group (×), $n$ = 7, LM 0.3% group (●), $n$ = 7, LM 1% group (▼), $n$ = 7. * and #, $p < 0.05$ when compared with the Control and GVHD groups, respectively.

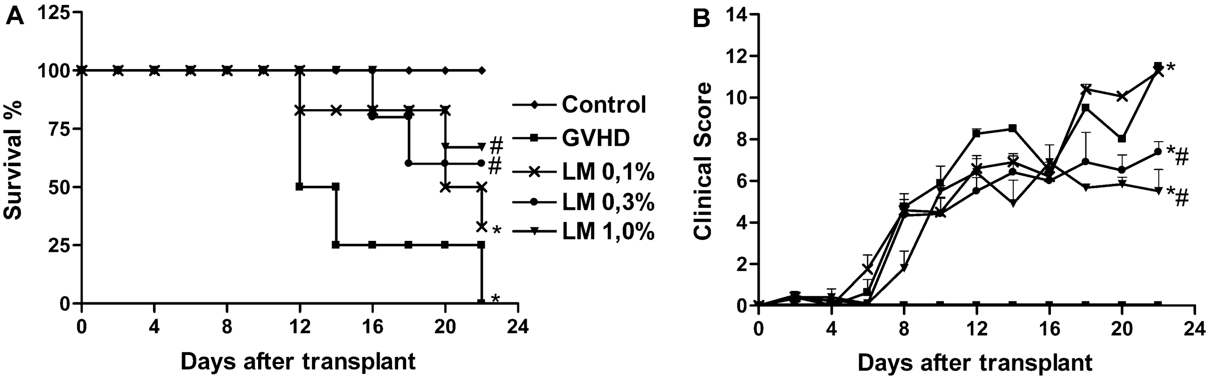


**Figure S2.** *Lithothamnion muelleri* extract (LM) treatment did not reduce the concentrations of IFN-γ and CCL2 in the liver of mice subjected to GVHD. GVHD was induced by the transfer of splenocytes from C57BL/6J donors to B6D2F1 mice. Mice that received syngeneic (B6D2F1) splenocytes did not develop disease and were considered the Control group. LM (1% in the diet, w/w) was offered in the diets of recipient mice from 1 day before transplantation until the experimental endpoint. At 20 days after transplantation, the mice were sacrificed and the concentrations of IFN-γ (**A**) and CCL2 (**B**) in the liver homogenates were evaluated by ELISA. The results are shown as the mean ± SEM ($n$ = 5); * $p < 0.05$ when compared with the Control group.

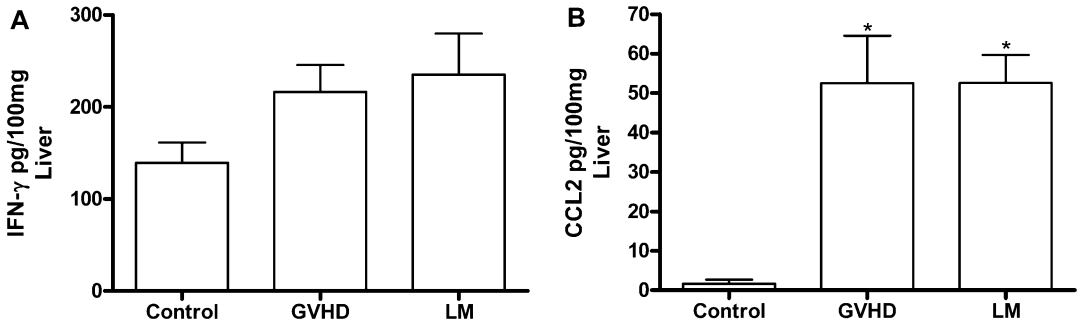

**Figure S3.** Calcium carbonate ($CaCO_3$) is not involved in the *Lithothamnion muelleri* extract-mediated protection of mice subjected to GVHD. GVHD was induced by the transfer of splenocytes from C57BL/6J donors to B6D2F1 mice. Mice that received syngeneic (B6D2F1) splenocytes did not develop disease and were considered the Control group. LM (1% in the diet, w/w; LM group) or $CaCO_3$ (0.9% in the diet, w/w; $CaCO_3$ group) was offered in the diets of recipient mice from 1 day before transplantation until the experimental endpoint. After the induction of GVHD, the mice were evaluated every 2 day for body weight (**A**) and GVHD clinical scores (**B**). The results are shown as the mean ± SEM. Control group (♦), $n = 6$; GVHD group (■), $n = 7$; LM group (▼), $n = 7$ and $CaCO_3$ group (○), $n = 7$. * and #, $p < 0.05$ when compared with the Control and GVHD groups, respectively.

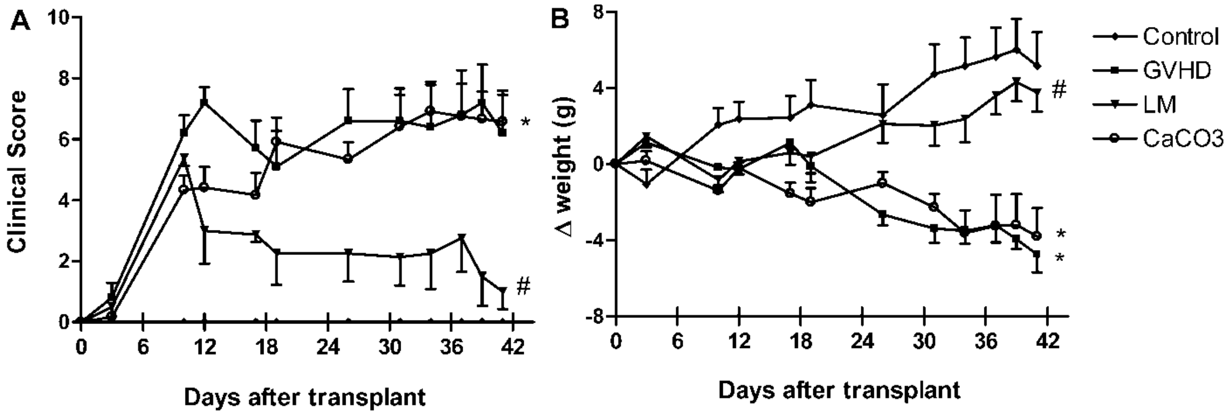


**Figure S4.** *Lithothamnion muelleri* extract (LM) treatment protects mice in a model of severe GVHD. GVHD was induced by the transfer of $3 \times 10^7$ splenocytes and $1 \times 10^7$ bone marrow cells from C57BL/6J donors into B6D2F1 mice that had been irradiated with a lethal dose for bone marrow depletion. Mice that received syngeneic (B6D2F1) splenocytes did not develop disease and were considered the Control group. LM (1% in the diet, w/w) was offered in the diets of recipient mice from 1 day before transplantation until the experimental endpoint. After the induction of GVHD, the mice were evaluated every 2 day for survival (**A**), clinical scoring (**B**) and body weight (**C**). The results are shown as the mean ± SEM. Control group (♦), $n = 6$; GVHD group (■), $n = 7$ and LM group (▼), $n = 7$. * and #, $p < 0.05$ when compared with the Control and GVHD groups, respectively.

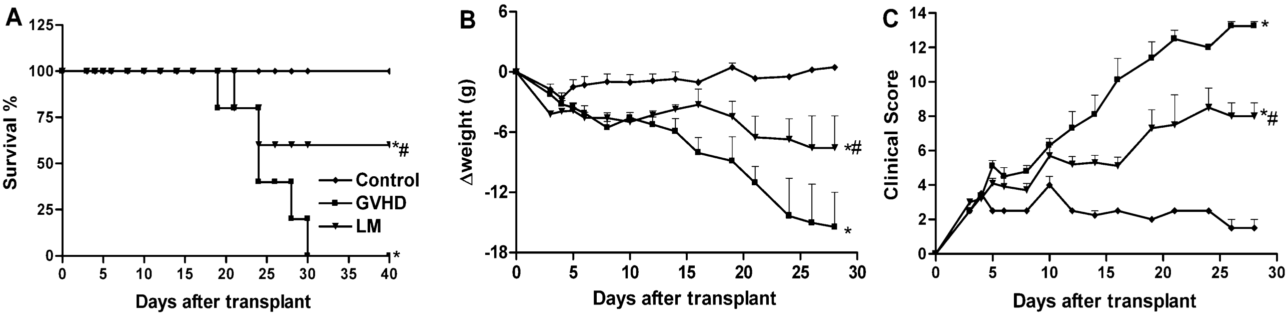

Supplement: Supplementary File 1 — Supplementary Information (PDF, 135 KB) [file marinedrugs-11-02595-s001.pdf]
